# Construction and evaluation of a prognostic prediction model based on the mEGOS score for patients with Guillain-Barré syndrome

**DOI:** 10.3389/fneur.2023.1303243

**Published:** 2023-11-30

**Authors:** Gaojie Xue, Yani Zhang, Ruochen Wang, Yue Yang, Huihui Wang, Jiangping Li, Xuexian He, Qing Zhang, Xiao Yang

**Affiliations:** ^1^Department of Neurology, General Hospital of Ningxia Medical University, Yinchuan, China; ^2^School of Public Health and Management, Ningxia Medical University, Yinchuan, China; ^3^Department of Cerebrospinal Fluid Laboratory, General Hospital of Ningxia Medical University, Yinchuan, China

**Keywords:** Guillain-Barré syndrome, mEGOS score, albumin, fasting plasma glucose, prognosis

## Abstract

**Background:**

Guillain-Barré syndrome (GBS) is an immune-mediated acute peripheral neuropathy in which up to 20% patients remain unable to walk independently after 6 months of onset. This study aimed to develop a clinical prognostic model based on the modified Erasmus GBS Outcome Score (mEGOS) for predicting the prognosis of GBS patients at 6 months of onset.

**Methods:**

The clinical data of 201 GBS patients were retrospectively analyzed. According to the GBS disability score (GBS-DS) at 6 months of onset, patients were divided into a good prognosis group (GBS-DS <3 points) and a poor prognosis group (GBS-DS≥3 points). Univariate and multivariate analysis was used to screen out independent risk factors for poor prognosis, and a prediction model was accordingly constructed for GBS prognosis.

**Results:**

The mEGOS score, serum albumin (ALB) and fasting plasma glucose (FPG) were independent risk factors for poor prognosis in patients with GBS, and the above risk factors were used to construct a prognostic model of mEGOS-I and a nomogram. The receiver operating characteristic (ROC) curve showed that the area under curve (AUC) of mEGOS-I at admission and at 7 days of admission to predict poor prognosis at 6 months of GBS onset was 0.891 and 0.916, respectively, with sensitivities of 82.7% and 82.6% and specificities of 86.5% and 86.6%, respectively. Decision curve analysis showed that the nomogram had a very high clinical benefit.

**Conclusion:**

To our knowledge, this is the first report of the construction of a prognostic prediction model based on the mEGOS score, ALB, and FPG that can accurately and stably predict the prognosis of GBS patients at 6 months of onset.

## 1 Introduction

Guillain-Barré syndrome (GBS) is an autoimmune-mediated acute inflammatory peripheral neuropathy. The characteristic clinical feature includes acute onset, and clinical symptoms usually peak in about 2 weeks, manifested by multiple nerve roots and peripheral nerve damage (1–3). With the widespread use of intravenous immunoglobulins (IVIG) and plasma exchange (PE), the mortality of GBS has now decreased compared to earlier, but there are still large differences in patient recovery and clinical outcomes. It has been reported that the mortality and disability rate of GBS patients can reach 7% and 20%, respectively, and uncomfortable symptoms such as paresthesia, pain, and fatigue can be as high as 40% (2, 4–6), which seriously affects their work efficiency and quality of life. Although most patients with GBS have received unified standardized treatment, with further understanding of the heterogeneity of clinical manifestations and prognosis of GBS, it is increasingly necessary to individualize and refine the treatment of patients in clinical practice, which makes early prognosis judgment more important. For example, IVIG pulse therapy is currently the standard treatment for GBS, but studies have shown that for some GBS patients who may have a poor prognosis, the use of PE combined with IVIG “zipper method” treatment can reduce both the mortality and degree of nerve defect and shorten the length of hospital stay (7). These depend on making reasonable disease trends and prognosis judgments for patients at an early stage of the disease, because this will help to formulate individualized treatment plans early, reduce unnecessary medical resource consumption, and reduce the mortality rate and disability rate of GBS.

GBS prognosis score is easy to use and is commonly applied for prognostic judgment in clinical practice. In 2007, Van Koningsveld et al. (8) established a simple clinical Erasmus GBS Outcome Score (EGOS) to assess patient prognosis, because the GBS prognosis score model can only evaluate the prognosis based on the GBS disability score at 2 weeks of onset, and is not suitable for identifying patients with poor prognosis in the early stage of the disease. In 2011, Walgaard et al. (9) established a modified EGOS (mEGOS) scoring system based on the EGOS model, using which a more accurate prognostic prediction could be obtained by scoring on day 7 of hospital admission. However, some scholars have suggested that patients are at different stages of disease onset upon admission, which may affect the score, thereby affecting the prediction outcome. Moreover, the score includes few predictors [only age, history of prodromal diarrhea, Medical Research Counsel (MRC) 3 clinical features] and is strongly subjective. Thus, some researchers have proposed that the scoring system should be improved by combining objective indicators such as biological markers to improve the overall prediction performance (10). With the continuous progress of related research at home and abroad in recent years, many biological markers have been confirmed to be related to the prognosis of GBS, such as serum anti-GD1a antibody IgG, anti-GM1 antibody IgG, serum neurofilament light chain, and glial fibrillary acidic protein (11–14). However, at present, the above test items have not been widely used in clinical practice, which limits their application in the prognosis and diagnosis of GBS. Neutrophils (NEU), platelets (PLT) and lymphocytes (LYM) are not only involved in the inflammatory and immune processes of the body, but are also markers of systemic inflammatory response. Albumin (ALB), fasting plasma glucose (FPG), neutrophil-to-lymphocyte ratio (NLR), and platelet-to-lymphocyte ratio (PLR) are routine clinical tests that have some predictive value on the state of inflammatory activity in inflammatory diseases, which has been demonstrated in several literatures. GBS is an autoimmune-mediated acute inflammatory peripheral neuropathy. It has been found that the above indicators have some predictive value on the prognosis of GBS (15–17).

Therefore, based on the mEGOS score, we combined ALB, FPG, NLR, PLR, and other routine clinical testing parameters to predict the adverse outcome of GBS and established a new prognostic prediction model—mEGOS-I—which showed better predictive power than the mEGOS score.

## 2 Materials and methods

### 2.1 Study population

The clinical and demographic data of 201 GBS patients the General Hospital of Ningxia Medical University from July 2015 to January 2022 were retrospectively collected. All patients received standard medical treatment, including immunotherapy, supportive care, and prophylactic treatment for complications following admission. The inclusion criteria were as follows: (1) fulfill the standard diagnostic criteria of GBS (18) based on the assessment by neurologists, and (2) age ≥18 years old. The exclusion criteria were patients with (1) incomplete clinical data (refers to those who have not completed serological biomarker testing or have incomplete serological biomarker testing within 1 week of admission); (2) chronic GBS; (3) Miller–Fisher syndrome; and (4) those lost to follow-up after 6 months of onset. This study was approved by the Medical Science Research Ethics Committee of the General Hospital of Ningxia Medical University and was conducted in accordance with the ethical guidelines of the 1975 Declaration of Helsinki. Given the retrospective nature of this study, the need for written informed consent was waived by the ethics committee.

### 2.2 Clinical features and laboratory indicators

Clinical features and laboratory indicators for each patient were obtained from the electronic medical records. Clinical features included patients' age and sex; season of onset; history of prodromal infection; MRC score at admission and day 7 of admission; mEGOS score at admission and day 7 of admission; and GBS disability score (GBS-DS) at admission, during peak disease, and at 6 months of onset. The biological markers (ALB, FPG, NEU, LYM, PLT, NLR, PLR) first measured after admission of the patient were collected and usually tested the day after admission. All patients were selected from baseline by uniformly trained neurologists and were followed up at 6 months of GBS onset. The results were recorded in detail, and all patient data were analyzed anonymously.

According to GBS-DS, patients were divided into a good prognosis group (GBS-DS <score of 3) and a poor prognosis group (GBS-DS≥ score of 3). Poor prognosis was defined as the inability to walk independently at 6 months of onset.

### 2.3 Evaluation tool

The mEGOS score included the following three items: (1) age ≤ 40 years: 0 points, 41–60 years: 1 point, > 60 years: 2 points; (2) without preceding diarrhea: 0 points, with preceding diarrhea: 1 point; and (3) MRC score: the mEGOS score at admission was calculated according to the MRC score at the time of admission, which was recorded as 0 points at 51–60 points, 2 points at 41–50 points, 4 points at 31–40 points, and 6 points at ≤ 30 points. The mEGOS score on the 7th day of admission was calculated according to the MRC score at 7 days of admission, namely 51–60 points was 0 points, 41–50 points was 3 points, 31–40 points was 6 points, and ≤ 30 points was 9 points. The total mEGOS score was between 0 and 9 points at the time of admission, and the total mEGOS score on the 7th day of admission was between 0 and 12 points. Peak disease was defined as the highest GBS-DS score or the lowest MRC sum score. mEGOS assessments were performed at the time of admission and on the 7th day of admission.

MRC score: In six groups of bilateral shoulder abduction, forearm flexion, wrist extension, thigh flexion, knee extension, and foot dorsiflexion, each group was graded from 0 to 5, and the total MRC score was 0 (tetraplegia) to 60 (normal muscle strength). A higher score indicates stronger muscle strength of the patient. The MRC score of an individual muscle group ranged from 0 to 5 points:

0 points: complete paralysis, no muscle contraction;
1 points: muscles can contract, but cannot produce movement;
2 points: limbs can move on the bed surface, but cannot resist their own gravity;
3 points: the limb can resist gravity to leave the bed, but not against resistance;
4 points: limbs can against resistance, but not completely;
5 points: normal strength.

The GBS-DS standards are as follows:

0 points: completely normal;
1 points: mild symptoms or signs, but able to run;
2 points: can walk independently ≥10 m without help, but cannot run;
3 points: can walk 10 m with help;
4 points: bedridden or requiring wheelchair;
5 points: need assisted ventilation;
6 points: death.

### 2.4 Statistical analysis

Statistical analysis was performed using SPSS26.0 (IBM Corporation; Armonk, NY, USA). Continuous variables conforming to the normal distribution were expressed by mean ± standard deviation, and the *t*-test of two independent samples was used for comparison between the two groups. The non-normally distributed continuous variables were expressed as medians (interquartile ranges), and the Mann–Whitney test was used for comparison between the two groups; categorical variables were expressed by frequency (composition ratio%), and the chi-square test was used for comparison between groups. Using univariate and multivariate analysis, independent risk factors related to the prognosis of GBS patients were screened to construct a clinical prediction model. Receiver operating characteristic (ROC) curve was applied to determine optimal cut-off values and assess the predictive ability of prognostic indicators. Then, The ROC curve of the model was drawn, and the area under curve (AUC) was used as the predictive value evaluation index to compare the predictive abilities of the mEGOS and mEGOS-I scores. Finally, the model is displayed in the form of a nomogram, and the risk prediction probability of poor prognosis of patients can be obtained by calculating the score. Hosmer–Lemeshow test was used to determine the goodness-of-fit of the model, and the decision curve analysis (DCA) was drawn to evaluate the clinical validity. A two-sided *P* < 0.05 was considered to indicate statistically significant differences.

## 3 Results

### 3.1 Demographic, clinical, and laboratory characteristics of the study population

In total, 303 GBS patients were included and 102 were excluded. The reasons for exclusion were chronic inflammatory demyelinating polyradicular neuropathy (*n* = 23), Miller–Fisher syndrome (*n* = 17), incomplete clinical data (*n* = 6), age <18 years (*n* = 19), and lost to follow-up at 6 months of onset (*n* = 37). Ultimately, 201 patients with GBS were included in the analysis. According to the prognosis of 6 months at onset, 149 patients were classified in the good prognosis group and 52 in the poor prognosis group. Poor prognosis most commonly occurred in patients aged >60 years, followed by patients aged 41–60 years, and the difference between groups was statistically significant (*P* < 0.05). The GBS-DS and FPG of the poor prognosis group were higher than those of the good prognosis group (*P* < 0.05). The MRC score, mEGOS score, and ALB of the poor prognosis group were lower than those of the good prognosis group (*P* < 0.05). There was no statistically significant difference with respect to sex distribution, season of onset, and history of antecedent infection between the two groups (*P* > 0.05) (Table 1).

**Table 1:** Comparison of baseline characteristics between good and poor prognosis group in GBS patients.

	**Good outcome (*N =* 149)**	**Poor outcome (*N =* 52)**	***P-*value**
**Sex**, ***n*** **(%)**			
Male	83 (55.7%)	30 (57.7%)	0.931
Femal	66 (44.3%)	22 (42.3%)	
**Age (years)**, ***n*** **(%)**			
≤ 40	43 (28.9%)	8 (15.4%)	0.002
41–60	65 (43.6%)	16 (30.8%)	
>60	41 (27.5%)	28 (53.8%)	
**Season**, ***n*** **(%)**			
Spring	26 (17.4%)	13 (25.0%)	0.093
Summer	43 (28.9%)	21 (40.4%)	
Autumn	46 (30.9%)	8 (15.4%)	
Winter	34 (22.8%)	10 (19.2%)	
**Preceding infection**, ***n*** **(%)**			
Absence	73 (49.0%)	31 (59.6%)	0.279
URTI	45 (30.2%)	9 (17.3%)	
Diarrhea	17 (11.4%)	8 (15.4%)	
Other	14 (9.4%)	4 (7.7%)	
GBS-DS on admission, median (IQR)	3.00 (2.00, 4.00)	4.00 (4.00, 4.00)	<0.001
GBS-DS during peak disease, median (IQR)	3.00 (3.00, 4.00)	4.00 (4.00, 5.00)	<0.001
MRC score on admission, median (IQR)	48.00 (38.50, 58.00)	29.50 (12.00, 44.25)	<0.001
MRC score at day 7 of admission, median (IQR)	54.00 (45.00, 60.00)	23.50 (10.25, 40.00)	<0.001
mEGOS on admission, median (IQR)	3.00 (1.00, 4.00)	6.50 (4.25, 8.00)	<0.001
mEGOS at day 7 of admission, median (IQR)	2.00 (1.00, 5.00)	9.00 (6.25, 11.00)	<0.001
NLR, median (IQR)	2.50 (1.85, 3.71)	5.20 (2.72, 12.32)	0.002
PLR, median (IQR)	140.78 (114.16, 199.51)	213.30 (128.70, 253.75)	0.004
ALB (g/L), median (IQR)	42.93 (38.10, 45.80)	34.90 (32.13, 41.78)	<0.001
FPG (mmol/L), median (IQR)	5.31 (4.89, 6.03)	6.60 (5.85, 9.60)	<0.001

GBS, Guillain-Barré syndrome; URTI, upper respiratory tract infection; IQR, interquartile range; GBS-DS, GBS disability score; MRC, Medical Research Council; mEGOS, modified Erasmus GBS Outcome Score; NLR, neutrophil to lymphocyte ratio; PLR, platelet to lymphocyte ratio; ALB, albumin; FPG, fasting plasma glucose.

### 3.2 Identification of NLR, PLR, ALB and FPG optimal cut-off values

ROC curves were used to calculate the optimum cut-off values for NLR, PLR, ALB, and FPG (Table 2). The AUCs were 0.718, 0.661, 0.749, and 0.758, respectively, and the optimal cut-off values were 3.47, 197.55, 36.05, 5.96, respectively. Patients were divided according to the optimal cut-off values of NLR, PLR, ALB and FPG into a low NLR group (≤ 3.47) and high NLR group (>3.47), low PLR group (≤ 197.55) and high PLR group (>197.55) group, low ALB group (≤ 36.05) and high ALB group (>36.05), and low FPG group (<5.96) and high FPG group (≥5.96).

**Table 2:** ROC curve analysis of ALB, FPG, NLR, PLR predicts prognosis in GBS patients.

	**AUC**	**95%CI**	**Cut off value**	***P-*value**
NLR	0.718	0.628–0.808	3.47	<0.001
PLR	0.661	0.569–0.753	197.55	0.001
ALB	0.749	0.668–0.831	36.05	<0.001
FPG	0.758	0.683–0.834	5.96	<0.001

NLR, neutrophil to lymphocyte ratio; PLR, platelet to lymphocyte ratio; ALB, albumin; FPG, fasting plasma glucose; ROC, The receiver operating characteristic; AUC, The area under curve; CI, Confidence interval.

### 3.3 Multivariate logistic regression analysis with poor prognosis of GBS patients

To avoid the problem of multicollinearity, mEGOS score, NLR, PLR, ALB, and FPG were finally included as independent variables, and poor prognosis at 6 months of onset was used as the outcome variable. Multivariate logistic regression analysis was carried out using the stepwise backward regression likelihood method. The results showed that mEGOS score, ALB, and FPG were independent predictors of poor prognosis at 6 months of onset of GBS patients (*P* < 0.05), and the above risk factors were used to construct a GBS prognostic prediction model mEGOS-I (Table 3).

**Table 3:** Multivariable logistic regression analysis for the construction of clinical prediction models.

	**OR**	**95%CI**	***P-*value**
**mEGOS-I-OA**			
mEGOS-OA	1.519	1.267~1.821	<0.001
ALB ≤ 36.05	5.759	2.415~13.735	<0.001
FPG≥5.96	5.282	2.275~12.264	<0.001
**mEGOS-I-D7**			
mEGOS-D7	1.456	1.275~1.663	<0.001
ALB ≤ 36.05	4.847	1.940~12.112	0.001
FPG≥5.96	5.624	2.291~13.806	<0.001

OA, on admission; D7, day 7 of admission; ALB, albumin; FPG, fasting plasma glucose; OR, Odds ratios; CI, Confidence interval.

### 3.4 The ROC curve of the GBS prognosis prediction model

**Figure 1:**
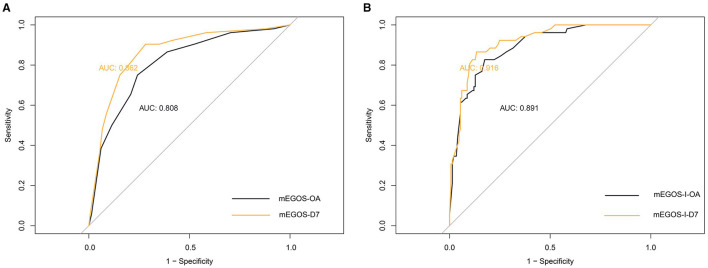
The ROC curves of the mEGOS score and the mEGOS-I score. **(A)** mEGOS **(B)** mEGOS-I. OA, on admission; D7, day 7 of admission.

Next, the predictive value of mEGOS score and mEGOS-I score on the prognosis of GBS patients was evaluated by analyzing the AUC values. The AUC under the curve for predicting the incidence of poor prognosis by mEGOS score at admission and 7 days of admission was 0.808 and 0.862, respectively. The AUC for predicting the incidence of poor prognosis by mEGOS-I score at admission and 7 days of admission was 0.891 and 0.916, respectively. The results showed that the mEGOS-I score had better discriminating ability than the mEGOS score (Figure 1).

### 3.5 Draw the nomogram of the prognostic prediction model of GBS

**Figure 2:**
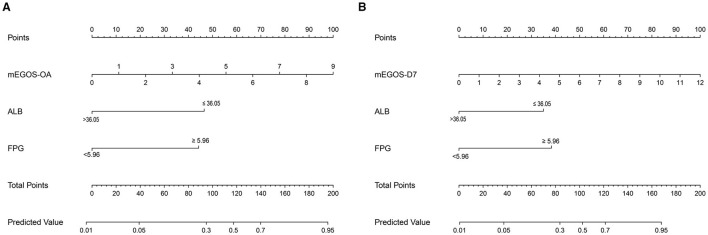
Nomogram predicting the prognosis of GBS patients at 6 months of onset. **(A)** mEGOS-I-OA, **(B)** mEGOS-I-D7. OA, on admission; D7, day 7 of admission; ALB, albumin; FPG, fasting blood glucose.

According to the results of multivariate analysis, the above independent influencing factors were included in the model as predictors, and the nomogram model was drawn to predict the prognosis of GBS patients. The total score of each variable in the graph was added to predict the incidence of adverse outcomes in GBS patients (Figure 2).

### 3.6 GBS prognosis prediction model calibration and clinical practicability evaluation

**Figure 3:**
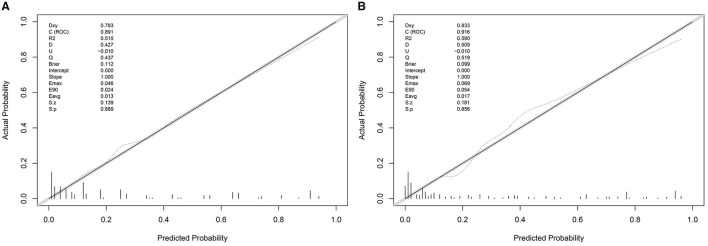
The calibration curve of the nomogram model. **(A)** mEGOS-I-OA, **(B)** mEGOS-I-D7. OA, on admission; D7, day 7 of admission.

**Figure 4:**
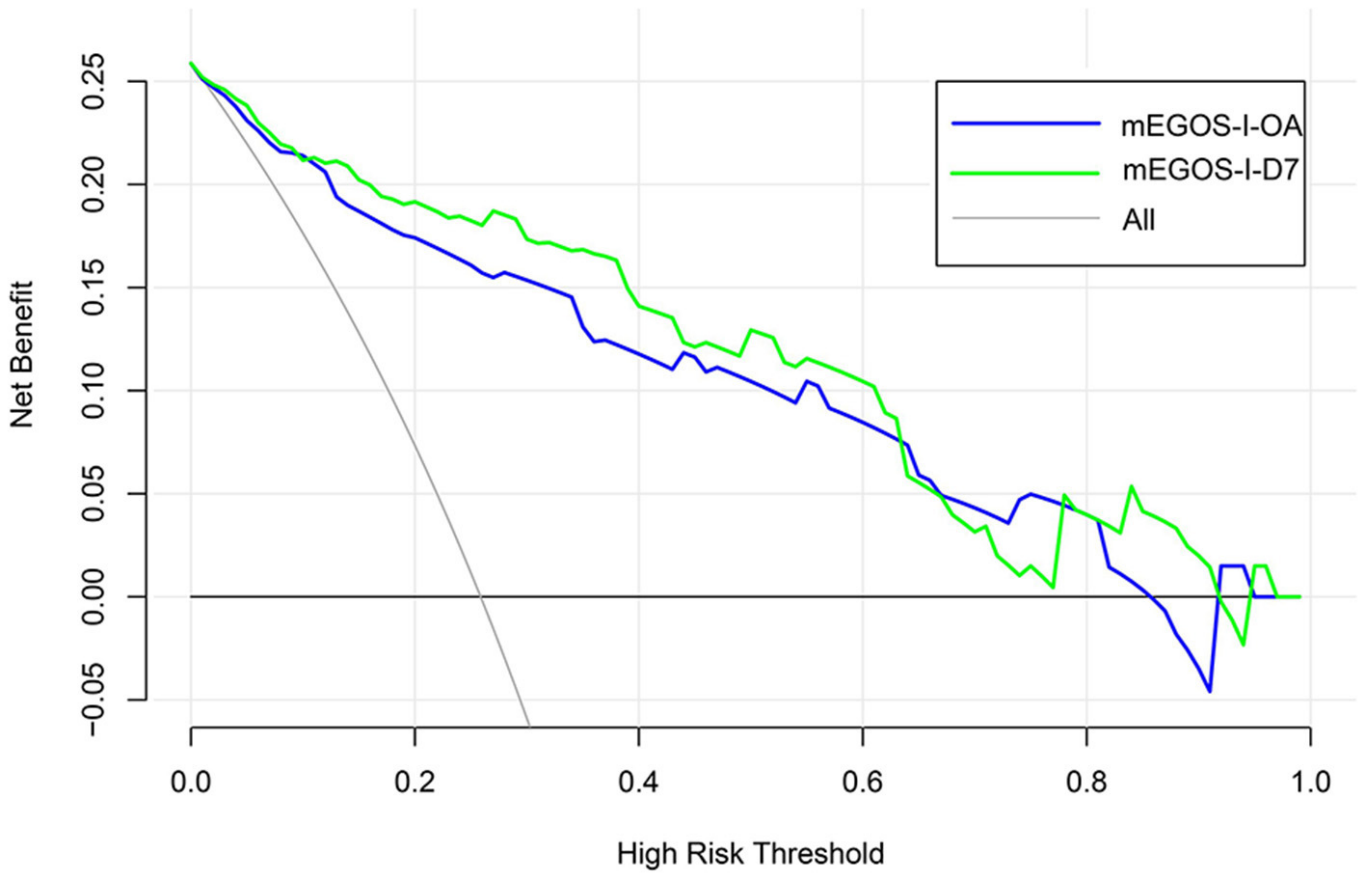
Decision curves of the nomogram model. OA, on admission; D7, day 7 of admission.

The Hosmer–Lemeshow goodness-of-fit test was used to evaluate the calibration degree of the mEGOS-I prediction model, and the results showed that the *P*-values were 0.9622 and 0.7501 at admission and 7 days of admission, respectively, and the *P*-values of both sets were > 0.05, indicating that the model was well calibrated (Figure 3). In addition, the clinical utility of the nomogram model was further evaluated by plotting decision curves, and the results showed that the model predicted GBS prognosis with a high net benefit and good clinical utility (Figure 4).

## 4 Discussion

This study explored the risk factors influencing the prognosis of GBS patients by retrospective analysis. The results of the multivariate logistic regression analysis showed that the mEGOS score, ALB, and FPG were the independent risk factors for predicting the prognosis of GBS (*P* < 0.05). Based on the above risk factors, we established a new GBS prognosis prediction model to improve the accuracy of adverse outcome risk prediction and facilitate the formulation of accurate and individualized treatment decisions in the early clinical stage.

The results of this study showed that the mEGOS score is an independent risk factor for GBS prognosis, and the mEGOS score at 7 days of admission had a higher predictive value than at admission, which is generally consistent with previous studies (11, 19). However, the predictive value of mEGOS remains to be further discussed. Papri et al. (10) validated the predictive value finding of mEGOS score on patient outcomes in a GBS cohort in Bangladesh, and correcting for mEGOS with existing predictors did not improve the discriminatory power of the model. A prospective, multicenter International GBS Outcomes Study (IGOS) (20) was externally validated for mEGOS scores in 1,500 GBS patients. The results showed that mEGOS underestimated the risk of adverse GBS outcomes for patients from Europe and North America, while for patients from Asia, mEGOS overestimated the risk of adverse GBS outcomes. The study re-estimated age, diarrhea history, and MRC score for calibrating the model, with only slight improvements in the AUC values of the area under the ROC curve. This suggests that to improve the accuracy of the prediction model, clinicians can try to introduce some more objective biological markers to optimize the model. Yamagishi et al. (11) used serum IgG anti-GD1a antibody combined with mEGOS score to predict the prognosis of GBS patients and found that the predictive power was higher than that of mEGOS score alone. However, the diagnostic value of anti-ganglioside antibodies in GBS has not been adequately demonstrated and therefore is not widely tested in GBS patients.

As a very common laboratory test item in clinical diagnosis and treatment, ALB is of great significance to assess the degree of infection, nutritional status, metabolic level, and immune response. Low serum ALB levels have been found to be an independent risk factor for the prognosis of GBS in adults (15). Studies by Zhang (21) and Ozdemir (22) have also confirmed that low serum ALB is associated with a poorer prognosis for GBS. This is likely because of the involvement of cellular redox imbalance and its resulting increase in free radicals in exacerbating demyelination and axonal damage in GBS patients (23, 24), while low serum ALB levels lead to a decrease in their ability to scavenge free radicals, resulting in irreversible neuronal loss. In addition, low ALB levels lead to a decrease in patients' immunity and resistance, which can easily cause complications such as infection, prolonging the course of the disease, and resulting in poor prognosis for GBS patients. The results of this study suggest that low ALB levels are an independent risk factor for GBS prognosis, consistent with the above findings. This indicates that clinicians should pay attention to the dynamic monitoring of ALB levels in GBS patients and strengthen nutritional support for patients with low ALB to enhance their disease resistance and improve the clinical outcomes.

Diabetes damages peripheral nerves through a variety of pathways (25, 26), and peripheral neuropathy occurs in up to 50% patients during disease progression (27). Bae et al. (28) compared the clinical features and electrophysiological outcomes of GBS patients with and without diabetes, and found that diabetes mellitus aggravated the clinical symptoms and peripheral nerve damage of GBS patients. Diabetes mellitus was an independent risk factor for poor prognosis of GBS patients at 3 months of onset. A growing body of studies have found that blood glucose levels are a good predictor of GBS prognosis (29, 30). Multicenter retrospective findings of Gong et al. (31) showed that glucose levels in blood and CSF were significantly associated with disability at admission, at disease peak, and at discharge, and was an independent risk factor for predicting short-term outcomes of GBS. The specific mechanism by which diabetes or hyperglycemia induction aggravates peripheral nerve damage is not fully understood, and may be related to the chronic inflammation caused by diabetes or hyperglycemia and insufficient blood supply to peripheral nerves. The ischemic state of neurons may lead to partial axonal damage or loss. Hyperglycemia may also lead to increased oxidative stress, induce protein and lipid damage, disrupt redox homeostasis, and ultimately lead to nerve damage (32–34). The state of systemic inflammatory response to GBS can lead to glucose metabolism disorders in patients, and the two may be causally related (35). The results of this study showed that high FPG levels are an independent risk factor for the poor 6-month prognosis in GBS patients, and that elevated blood glucose aggravated the dysfunction of GBS patients, delayed their motor recovery, and affected their outcome, which is generally consistent with the above findings. This suggests that active control of blood glucose levels may be an adjuvant therapy to improve the prognosis of GBS.

To our knowledge, this study is the first to construct the nomogram model—mEGOS-I—based on the mEGOS score combined with ALB and FPG to evaluate the prognosis of patients with GBS at 6 months of onset. The ROC curve analysis demonstrated that the mEGOS-I score had a better prognostic discriminatory ability compared with the mEGOS score. Calibration curve and decision curve analyses showed that the nomogram constructed in this study had good predictive stability and clinical utility in predicting the prognosis of GBS patients.

This study has some limitations. As our model was constructed based only on retrospective clinical databases with inevitable recall bias, some variables could not be introduced in the logistic regression analysis because of missing data such as electrophysiological subtypes, cranial nerve involvement, and autonomic nerve involvement. Moreover, as a single-center retrospective study, further multi-center, large-sample, prospective studies are needed to validate the predictive value of this model in its prognostic aspects.

## 5 Conclusion

In conclusion, the nomogram model mEGOS-I constructed in this study based on mEGOS score, ALB, and FPG has a good prognostic predictive value and can accurately and stably predict the prognosis of GBS patients at 6 months of onset.

## Data availability statement

The data analyzed in this study is subject to the following licenses/restrictions: The datasets supporting the conclusions of this article are available from the corresponding author upon reasonable request. Requests to access these datasets should be directed to cckk606@sina.com.

## Ethics statement

This retrospective study was approved by Review Board of General Hospital of Ningxia Medical University of Science and Technology. The studies were conducted in accordance with the local legislation and institutional requirements. Written informed consent for participation was not required from the participants or the participants' legal guardians/next of kin in accordance with the national legislation and institutional requirements.

## Author contributions

GX: Conceptualization, Data curation, Formal analysis, Methodology, Writing—original draft. YZ: Data curation, Writing—original draft. RW: Writing—review & editing. YY: Writing—review & editing. HW: Writing—review & editing. JL: Writing—review & editing. XH: Writing—review & editing. QZ: Supervision, Writing—review & editing. XY: Funding acquisition, Supervision, Writing—review & editing.
